# Methylboronic acid MIDA ester (ADM) as an effective additive in electrolyte to improve cathode electrolyte interlayer performance of LiNi_0.8_Co_0.15_Al_0.05_O_2_ electrode

**DOI:** 10.1038/s41598-023-36341-8

**Published:** 2023-06-20

**Authors:** Bo-Xun Chen, Sanjaya Brahma, Yu-Qi Chen, Po-Chia Huang, Chia-Chin Chang, Jow-Lay Huang

**Affiliations:** 1 Department of Materials Science and Engineering, National Cheng Kung University, Tainan, 701 Taiwan; 2 R & D Center for Li-Ion Battery, National University of Tainan, Tainan, 70005 Taiwan; 3 National Synchrotron Radiation Research Center (NSRRC), Hsinchu, 300 Taiwan; 4 Department of Greenergy, National University of Tainan, Tainan, 70005 Taiwan; 5 Hierarchical Green-Energy Materials (Hi-GEM) Research Center, National Cheng Kung University, Tainan, 701 Taiwan; 6 Center for Micro/Nano Science and Technology, National Cheng Kung University, Tainan, 701 Taiwan

**Keywords:** Electrochemistry, Batteries

## Abstract

We investigated the effectiveness of using methylboronic acid MIDA ester (ADM) as an additive in an electrolyte to enhance the overall electrochemical and material properties of an LNCAO (LiNi_0.8_Co_0.15_Al_0.05_O_2_) cathode. The cyclic stability of the cathode material measured at 40 °C (@ 0.2 C) showed an enhanced capacity of 144.28 mAh g^−1^ (@ 100 cycles), a capacity retention of 80%, and a high coulombic efficiency (99.5%), in contrast to these same properties without the electrolyte additive (37.5 mAh g^−1^, ~ 20%, and 90.4%), thus confirming the effectiveness of the additive. A Fourier transform infrared spectroscopy (FTIR) analysis distinctly showed that the ADM additive suppressed the EC-Li^+^ ion coordination (1197 cm^−1^ and 728 cm^−1^) in the electrolyte, thereby improving the cyclic performance of the LNCAO cathode. The cathode after 100 charge/discharge cycles revealed that the ADM-containing system exhibited better surface stability of the grains in the LNCAO cathode, whereas distinct cracks were observed in the system without the ADM in the electrolyte. A transmission electron microscopy (TEM) analysis revealed the presence of a thin, uniform and dense cathode electrolyte interface (CEI) film on the surface of LNCAO cathode. An operando synchrotron X-ray diffraction (XRD) test identified the high structural reversibility of the LNCAO cathode with a CEI layer formed by the ADM, which effectively maintained the structural stability of the layered material. The additive effectively inhibited the decomposition of electrolyte compositions, as confirmed by X-ray photoelectron spectroscopy (XPS).

## Introduction

Lithium ion batteries (LIBs) are considered integral to the future development of energy storage devices, for use not only in portable electronic gadgets (e.g. laptops and smart phones) but also in high-capacity devices (e.g. electric vehicles, energy storage systems), because they present several advantages such as their high energy density, good cyclability and safety. However, research into improving all these parameters typically depends on the individual components (the anode, cathode, separator, etc.) of LIBs along with the microstructure and composition of the batteries.

Cathode materials are an important component of an LIB that significantly influences its electrochemical properties and its impact on the overall performance of the device. Therefore, one of the aims of recent studies is to develop and modify environmentally friendly cathode materials in order to enhance energy density and cyclic stability. The most commonly investigated layered cathode materials are LiCoO_2_ and LiNiO_2_, which have a hexagonal structure. However, they are not without problems: the presence of Co in LiCoO_2_ comes with a high cost and toxicity, and side reactions between the electrolyte solution and Ni^4+^ produced during the delithiation of LiNiO_2_ lead to a lower output capacity^1^. Efforts to overcome these shortcomings have led to the development of an Ni-rich cathode material, the so-called LNCAO (LiNi_0.8_Co_0.15_Al_0.5_O_2_), that is available at a low cost, achieves a high capacity (200 mAh g^−1^), and delivers a high voltage^2–7^. However, surface degradation at high temperatures and high-voltage cycling during subsequent cycles usually leads to significant changes in the microstructure of the interface between the electrode and the electrolyte, which often reduces the cyclic stability and the rate capability of the cathode as well as the overall performance of the battery. In a way that is similar to a solid electrolyte interface (SEI) layer forming at the interface between an electrode and the electrolyte, a cathode electrolyte interface (CEI) layer is also generated at the cathode-electrolyte interface, and the stability of this layer is crucial to preventing the decomposition of the electrolyte. Furthermore, the generation of corrosive hydrogen fluoride (HF) and CO_2_ in electrolyte solutions containing the LiPF_6_ salt is another serious problem that occurs. Addressing issues related to the surface passivation of the cathode materials and the incorporation of additives into the electrolyte are some recent ideas being developed with the goal of enhancing the potential of the positive electrode.

Recently, additives in the electrolyte solution of an LIB have been used to control the formation and composition of a CEI layer in order to prevent the decomposition of the electrolyte and to enhance the cyclability of the LIB^8^. Several additives such as tris(trimethylsilyl)-phosphite (TTSPi)^9^, methylene methane disulfonate (MMDS), vinylene carbonate (VC), lithium bis(oxalate)borate (LiBOB), tributyl borate (TBB)^10^, and phenyl vinyl sulfone (PVS), as well as a combination of additives have proven beneficial to the high-temperature (55 °C) cyclic stability of LIBs by reducing impedance and the formation of undesired gases. Regarding efforts to counteract the adverse impact of high-voltage cycling, Kazzazi et al.^11^ noted improvements in the performance of LCP (LiCoPO_4_) and LNMO (LiNi_0.5_Mn_1.5_O_4_) cathodes when using a combination of TTSPi and bis(2,2,2-trifluoroethyl) carbonate (TFEC) at a cell voltage higher than 4.5 V. This combination of additives was highly effective in suppressing the degradation of the electrolyte, the generation of gases, and the dissolution of transition metals.

Boron-based compounds are widely considered suitable as electrolyte additives in order to enhance the performance of the cathode. For example, Xiao et al.^12^ added LiBOB (lithium bis(oxalate)borate) to an electrolyte solution of 1.0 M LiPF_6_ in an ethylene carbonate/ethyl methyl carbonate (EC/EMC) solvent blend. Doing so induced the formation of a dense and stable CEI layer (15 nm) which produced a very good electrochemical performance of the cathode material (Li_1.2_Mn_0.54_Co_0.13_Ni_0.13_O_2_ microspheres), with a stable capacity of 202 mAh g^−1^ @ 0.5C and a very good capacity retention of 96.4% after 100 cycles. Another relevant study was conducted by Huang et al.^10^ who investigated the effects of adding TBB (tributyl borate) to the electrolyte solution on the stability of the interface between the LNMO cathode and the electrolyte at 55 °C. The additive produced a thin, low-resistance film on the cathode surface that prevented the degradation of the carbonate solvent and inhibited the dissolution of the transition metal ions Ni and Mn from the LNMO. The additive TBB decomposed and participated in the formation of the CEI, which provided good protection and resistance against the oxidative decomposition of the electrolyte, and changed the local environment between the Ni and Mn ions on the surface of the LNMO cathode, leading to a more stable structure at the high voltage^13^.

**Figure 1:**
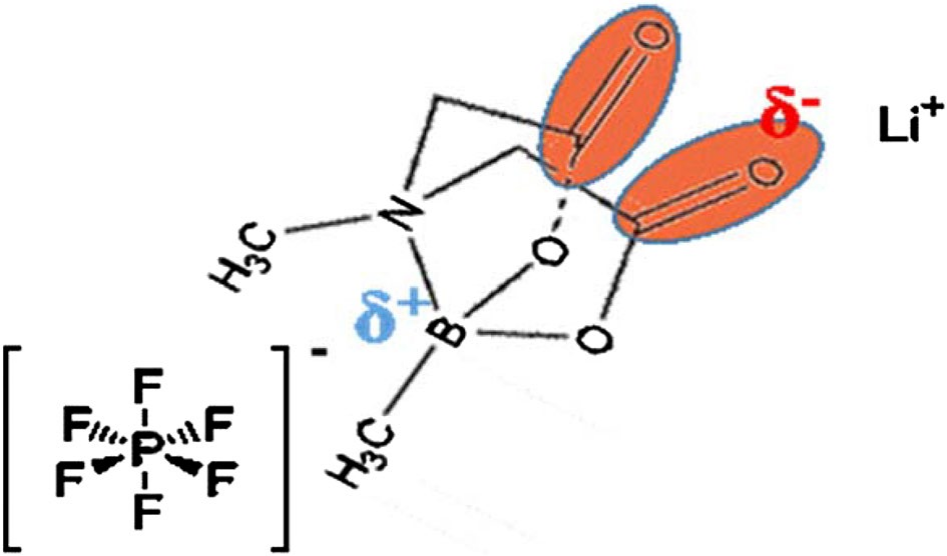
The molecular and electron affinity labelled structure of ADM.

In this study, we explored the use of a methylboronic acid MIDA ester (ADM) (Fig. 1) as an additive in the electrolyte to enhance the cyclic stability, rate capability and overall electrochemical properties of an LNCAO (LiNi_0.8_Co_0.15_Al_0.05_O_2_) cathode. Methylboronic acid MIDA ester (ADM) is supposed to be a suitable additive for the electrolyte. Addition of ADM would influence the interaction with the ions such as Li^+^, PF_6_^−^ and EC in the electrolyte and avoid the decomposition of the organic solvents EC and DEC thereby modifying the composition of the CEI film and improving the structural stability of the cathode material. A detailed analysis of how the behavior of the ADM additive improved the performance of the LNCAO cathode was conducted.

## Experimental design

### Material preparation

**Figure 2:**
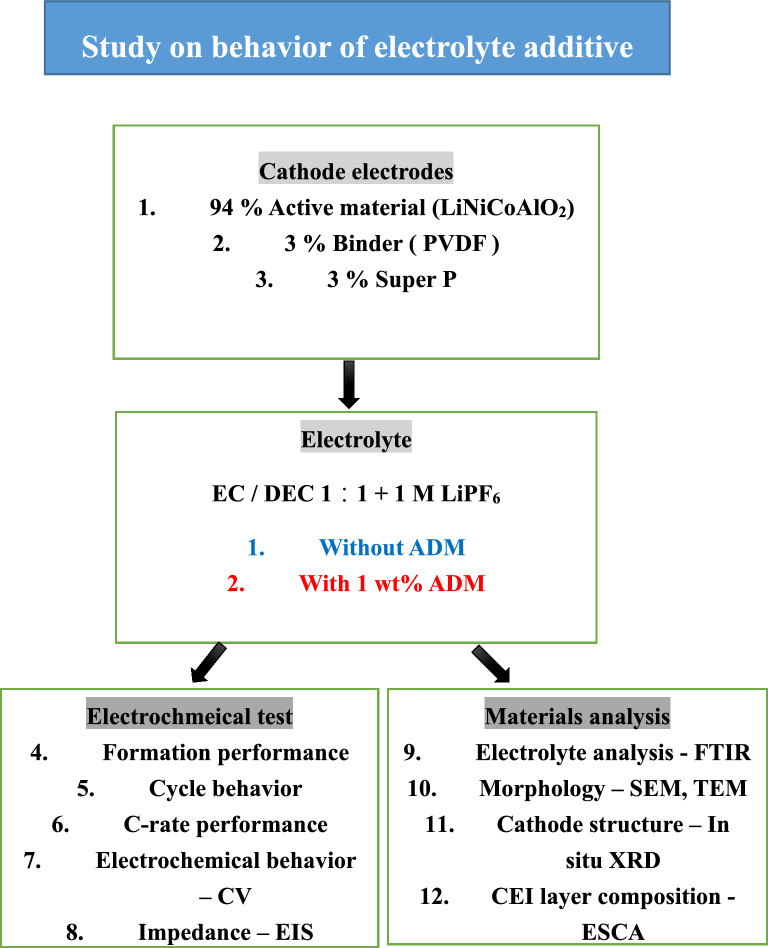
Framework of experiment.

In this study, the main active material in the cathode (LNCAO, Nihon Kagaku Sangyo Co., Japan) was prepared by creating a composite made of 93.5% active material, 1.5% conductive carbon (Super P, Timcal), 1% vapor grown carbon fiber (VGCF, Showa Denko K.K., Japan), and 4% binder polyvinylidene difluoride (PVDF, W1700, Kureha, Japan). Framework of the experiment is shown in Fig. 2.

### Material characterization

We investigated the microstructure of the cathode by means of high-resolution field emission scanning electron microscopy (HR-FESEM) and ultra-high resolution analytical electron microscopy (HR-AEM). The energy levels of the CEI layer composite were determined by means of electron spectroscopy for chemical analysis (ESCA). In-situ X-ray diffraction (In-situ XRD, National Synchrotron Radiation Research Center TLS 17A, NSRRC) was used to determine the crystal structure of the LNCAO, with X-rays emitted at a wavelength of 1.3208 Å, an angular speed of 10°(2θ)/min, and a 2θ angle of 0° to 60°. The composition and morphology of such a CEI film may be altered by the addition of an electrolyte, lithium salts, electrode materials, and electrolyte additives. In order to assess any changes in our battery, it was disassembled in a glove box. The cathode was then taken out, cleaned with DEC to remove any residual lithium salt, and left inside the glove box in an argon (Ar) atmosphere overnight to remove the electrolyte. Finally, it was sealed inside an aluminum foil pouch containing argon to avoid any atmospheric contamination before analysis.

### Electrochemical analysis

The charge/discharge performance of the LNCAO cathode was determined by using coin cells composed of lithium foil electrodes, a separator (Celgard, 2300), an electrolyte solution of 1 mol/dm^3^ of LiPF_6_/EC/diethyl carbonate (DEC) (1:1 wt%) with 1 wt% ADM (Hopax Chemicals Manufacturing Co, Taiwan). The charge/discharge performance of the cells was tested with a constant current between 2.5 and 4.2 V at 0.1C, and cycle life was tested for 100 cycles. Tests were conducted at different current rates (0.1–1C) to determine the capacity at high rates. Cyclic voltammetry (CV) measurements were taken at room temperature (25 °C) at a scan rate of 0.1 mV/s in the voltage range of 2.5–4.5 V. Electrochemical impedance spectroscopy was performed on the coin cells at open circuit voltage by coupling a potentiostat to an Autolab frequency response analyzer locked in an amplifier and an impedance phase analyzer. A sinusoidal amplitude modulation of ± 10 mV was used over a frequency range from 0.01 Hz to 1 MHz.

## Results and discussion

**Figure 3:**
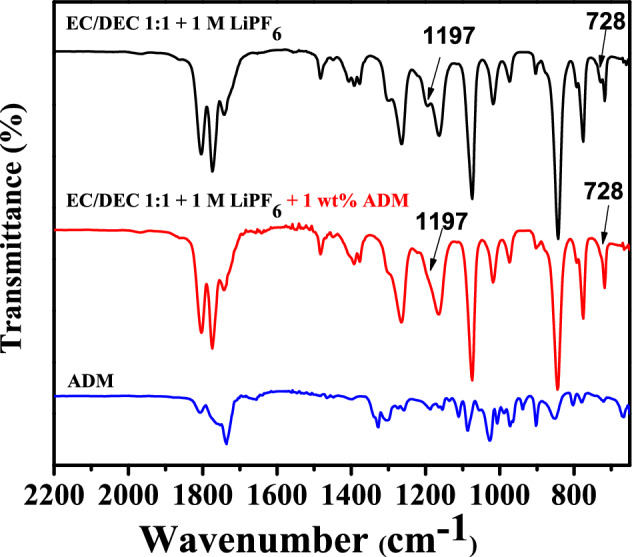
FTIR spectra of ADM, electrolyte system with 1 wt% ADM, and without additive electrolyte system (EC/DEC 1:1 + 1 M LiPF_6_).

Figure 3 shows the comparative FTIR analysis of the ADM alone, the ADM-containing electrolyte, and the electrolyte with the ADM additive. The absorption peaks falling within the ranges of 1736–1809 cm^−1^ and 972–1100 cm^−1^ was attributable to the C=O and C–O stretching vibrations (Table 1), representing the bonding of organic compounds. The intensity of the absorption peak for the ADM alone was relatively weaker than that for the electrolyte both with and without the ADM. The absorption peak for boron nitride (BN) bonding in ADM occured at 777–902 cm^−1^ and 1255–1329 cm^−1^^14–17^, and the peaks at 1086 cm^−1^ and 1135 cm^−1^ were the result of B–C and C–N vibrations, respectively^17^. The characteristic peaks related to boron occured at 1029 cm^−1^ for B–O–C bonding, and at 667 cm^−1^ and 1350 cm^−1^ for B–O asymmetric stretching peaks^18–20^. A careful analysis of the FTIR spectra showed clearly distinct absorption peaks at 1197 cm^−1^ and 728 cm^−1^ (EC-Li^+^ coordination^21^) for the electrolyte without ADM, whereas these peaks were absent for the additive-containing electrolyte, revealing the coordination effect of the Li^+^ ion in the electrolyte additive. Another absorption peak was seen for all the compounds within the range of 1736–1809 cm^−1^, corresponding to C=O stretching vibrations. The peaks at 1450–1480 cm^−1^, 1162–1302 cm^−1^, and 972–1100 cm^−1^ corresponded to C–H bending, O–C–O vibration peaks, and a C–O absorption peak, respectively. Finally, the peak at 840 cm^−1^ was the P–F characteristic peak of lithium phosphate^22–24^.

**Table 1:** FTIR data for the electrolyte and additive.

Wavenumber (cm^−1^)	Assignment	Functional group	Species
1805 1774	C=O stretch	O=C–O	EC
1741	C=O stretch	O=C–O	DEC
1809 1736	C=O stretch	O=C–O	ADM
1481	C–H sym scissor	CH_2_	EC
1448	C–H asym bend	CH_2_	DEC
1391	C–H wagg	CH_2_	EC
1302–1162	C–O stretch	O-C-O	DEC EC
1100–972	C–O stretch	C-O	DEC EC ADM
840	P–F stretch	PF_6_^−^	LiPF_6_
1329–1255, 902–777	h-BN	B-N	ADM
1187–1159	C–H bonds	C–H	ADM
1135	C–N stretch	C–N	ADM
1115–1089	B–C bonds	B–C	ADM
1029 667 and 1350	B–O–C bond B–O units	B–O–C B–O	ADM
1197 and 728	EC-Li^+^		

**Figure 4:**
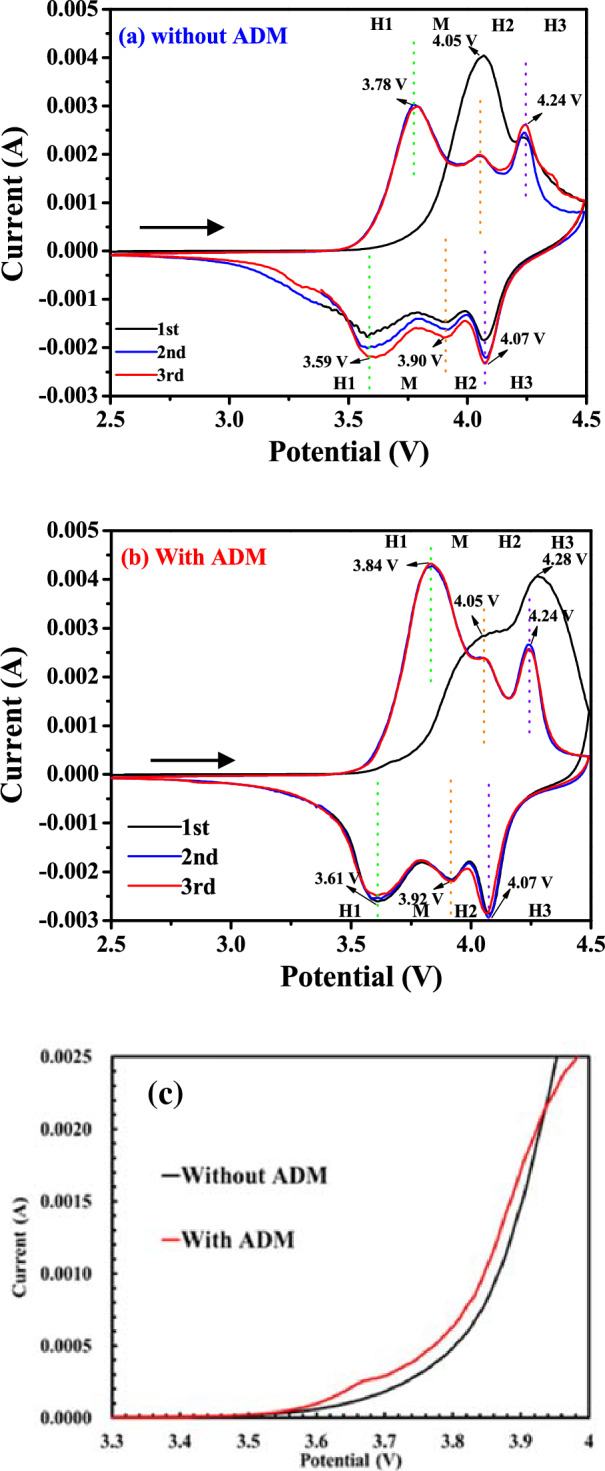
Cyclic voltammetry of NCA/Li half cells in 1 M LiPF_6_ dissolved in EC/DEC (1:1) (**a**) without ADM; (**b**) with 1 wt% ADM; (**c**) enlarge 3.3–4 V at 1st scan.

Cyclic voltammetry provided an accurate analysis of the electrochemical reactions and phase transitions occurring in electrode materials as well as the influence of the additives on the oxidation–reduction potential of LNCAO^25,26^. The reduction process involved a phase transformation from a hexagonal structure (H1) to a monoclinic structure (M) which then converted to a second (H2) and a third (H3) hexagonal phase at specific reduction peaks. Figure 4a,b shows the cyclic voltammogram of the cathode without and with the electrolyte additive. All the transition phases (H1, M, H2, H3) can be seen at the reduction peaks. The first oxidation peak at approximately 3.68 V for the ADM-containing system is small and broad but is not obvious when the electrolyte does not contain the additive as shown in Fig. 4c. This result shows that the ADM additive changed the process of the formation of the CEI film during the first cycle. In the first cycle, both electrolytes, with and without the ADM showed an irreversible oxidation peak at 4.05 V due to the conversion of Ni^3+^ to Ni^4+^ and the formation of the CEI film^26^. The intensity of the oxidation at this voltage was weak in the presence of the additive (Fig. 4b), which may have inhibited the conversion of Ni^3+^ to Ni^4+^ and the formation of a relatively stable CEI film. The redox peaks of the electrolyte with the additive during the second and third cycles overlaped, indicating good reversibility and stability of the CEI film. The transition from the H1 phase to the M phase between the second and third cycles was a result of the conversion of Ni^3+^ to Ni^4+^. The location of the oxidation peak for the ADM-containing system (at 3.84 V) was slightly higher than it is for the electrolyte without the additive (3.78 V) as shown in 2nd and 3rd cycles in Fig. 4. This indicated that the ADM facilitated the transition from the H1 phase to the M phase on the surface of the LNCAO cathode at high voltages, with the higher oxidation peak caused by the decrease in the cation mixing phenomenon^27^ which might lead to higher electrochemical activity. Cation mixing generally involves the displacement of the Ni^+2^ ions from the transition metal layer to the lithium layer during charge/discharge process or during sintering leading to partial reduction of Ni ions due to the non-stoichiometry thereby causing severe degradation of the NCA material and large capacity fading. The transition from the M phase to the H2 phase was a result of the compression of the c-axis caused by the excessive insertion of lithium ions. This compression effect, combined with the high oxidation number of the transition metal, stretched both the a-axis and the b-axis, which resulted in a substantial reduction in volume, causing the H2-to-H3 transformation^28^. Therefore, the ADM additive formed a CEI film on the surface of the LNCAO cathode, which stabilized the surface structure of the LNCAO, leading to the stabilization of its bulk structure.

**Figure 5:**
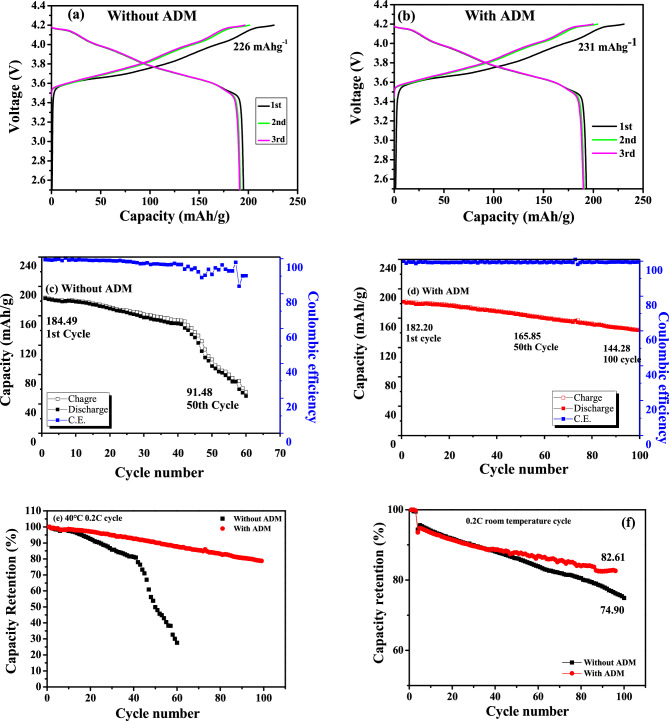
Voltage profile of LNCAO/Li half cells in 1 M LiPF_6_ dissolved in EC/DEC (1:1) (**a**) without ADM and (**b**) with 1 wt% ADM (0.1C), cycling performance and coulombic efficiency of LNCAO/Li half cells in 1 M LiPF_6_ dissolved in EC/DEC (1:1) at 40 °C (0.2C) (**c**) without ADM, (**d**) with 1 wt% ADM, and capacity retention with/without additive (**e**) 40 °C @ 0.2C, (f) 25 °C @ 0.2C.

The charge/discharge analysis (for the first three cycles) of the LNCAO cathode material (Fig. 5a,b) showed that the first charge capacity of the cathode (@ 0.1C) with the ADM (~ 231 mAh g^−1^) was slightly higher than that of the cathode without it (~ 226 mAh g^−1^). The discharge capacity and the CE of the cathode were slightly lower during the first three cycles with the ADM (193 mAh g^−1^, 83.6%) than without it (195 mAh g^−1^, 86.4%), which might be attributable to the initial chemical reactions happening on the electrode. Figure 5c,d shows the cyclic stability of the cathode (@ 0.2C) at 40 °C with and without the ADM in the electrolyte. After 100 charge/discharge cycles, the ADM-containing system (Fig. 5d) maintained a high capacity (~ 144.28 mAh g^−1^), a high retention (~ 80%), and a high CE (99.5%), whereas the system in which the electrolyte did not contain the additive (Fig. 5c) achieved a relatively low capacity (approximately ~ 91.48 mAh g^−1^ @50 cycles) and reduced retention rate (~ 50% which was a significant reduction of 40%), and a CE of 91% (Fig. 5e). Furthermore, the battery cycled at room temperature (25 °C) achieved 82.61% (with additive) and 75% (without additive) retention after 100 charge/discharge cycles (Fig. 5f). The electrochemical performance of our LNCAO cathode at 40 °C is comparable with room temperature data of LNCAO (163.00 mA h/g at first and 142.88 mA h/g at 100th cycle, current rate = 0.3C) prepared by using glycerol as solvent followed by heating at high temperature of 750 °C^29^ and F doped LNCAO^30^.

The analysis of the impedance occurring during different charge/discharge cycles which was used to examine the formation of the CEI layer after 3 and 100 cycles as shown in Figure S1 and the resistances of LNCAO electrode were simulated and listed in Table 2. It may be observed that the RCEI in the 3rd cycle with additive was slightly higher (5.15 Ohm) than without additive (3.96 Ohm) whereas the Rs and Rct were less. Similarly, at 100th cycle, the Rs was slightly higher (11 Ohm) with additive than without additive (3.75 Ohm) whereas the R_CEI_ and Rct were less. In any case, the overall resistance in LNCAO with additive was significantly lower as compared to without additive thereby leading much better Li ion transport in LNCAO with additive in the electrolyte. The ADM might exhibit stable impedance in LNCAO materials after 3 charge/discharge cycles, a stable CEI film was generated. The CEI film in the system without the ADM additive was uneven and thicker than the ADM-containing system where the film was relatively thin and uniform. This thin CEI layer was more conducive to the intercalation/de-intercalation of lithium ions, thus protecting the electrolyte from oxidation during cycling. A stable CEI film is very beneficial to the performance of a battery. Therefore, the ADM additive effectively modified the CEI film on the surface of the LNCAO cathode and inhibited the reaction between the LNCAO and the electrolyte, thereby increasing the cycle stability of the battery.

**Table 2:** Fitting of the circuit data on electrolyte systems without ADM and with 1wt% ADM after 3 and 100 cycles.

	R_S_ (Ω)		R_SEI_ (Ω)		R_ct_ (Ω)	
	Without ADM	With ADM	Without ADM	With ADM	Without ADM	With ADM
3rd	2.31	1.87	3.96	5.15	14.3	10.7
100th	3.75	11	16.2	10	163	75

**Figure 6:**
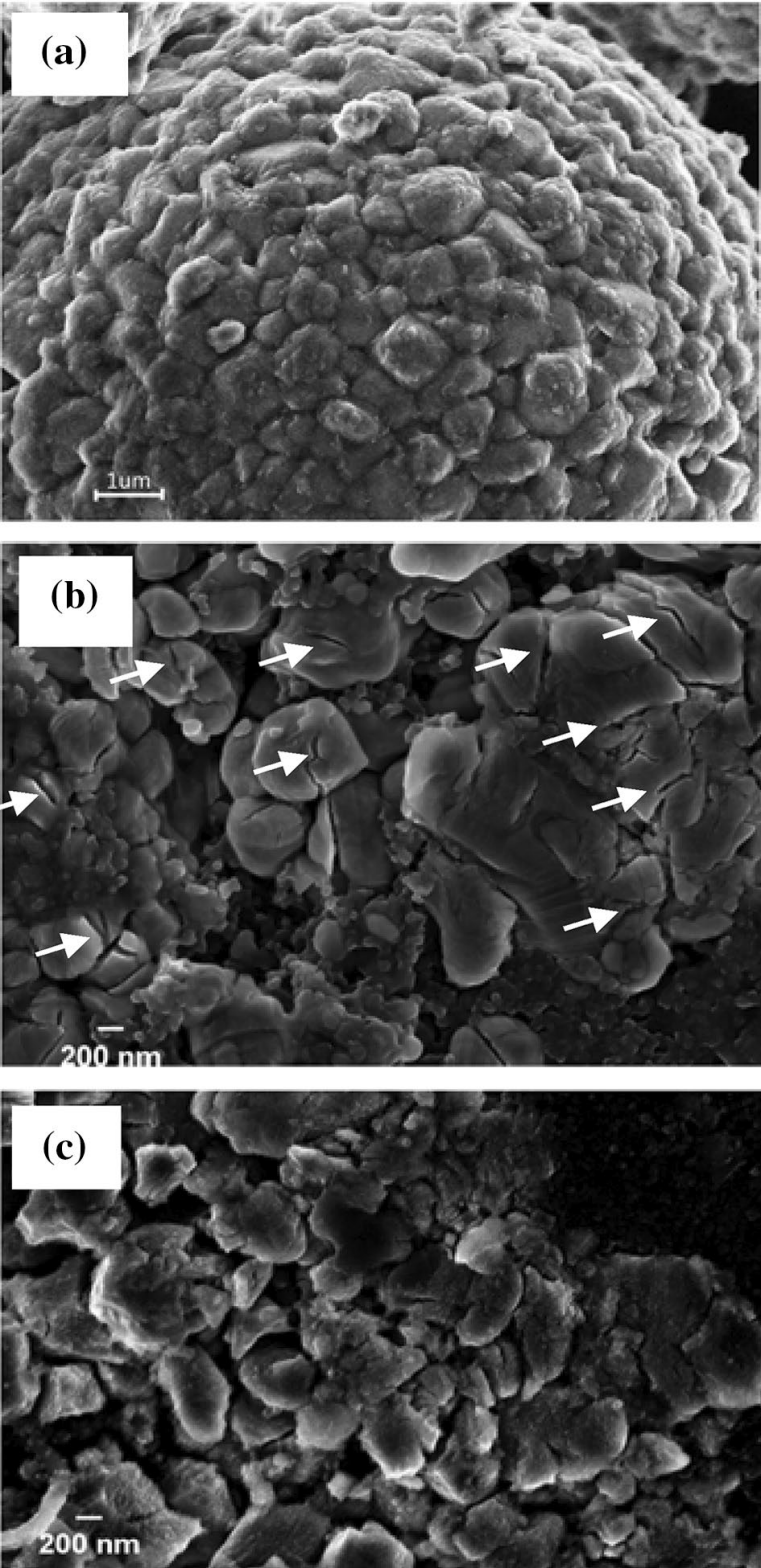
SEM images taken of the surface of the electrode: (**a**) a fresh LNCAO cathode, (**b**) a cycled cathode without ADM, and (**c**) a cycled cathode with 1 wt% ADM. The cycled electrodes were taken from coin cells after 100 cycles.

The morphology of the cathode before and after 100 charge/discharge cycles is presented in Fig. 6. Figure 6a shows the LNCAO cathode mixed with the PVDF binder before cycling, with a smooth microstructure containing well-connected particles. The conductive agent Super P can be seen as particles whose function was to increase the conductivity of the electrode. The SEM morphology of the cathode after 100 charge/discharge cycles when the electrolyte did not contain the additive (Fig. 6b) revealed the formation of cracks (white arrow marked) on the surface of the grain after several charge/discharge cycles, which did not appear on the uncycled electrode. The uncycled particles showed a granular morphology, whereas the cycled electrode had cracks and the surface layer was covered by a sediment with irregular particle shapes. When the ADM was added to the electrolyte (Fig. 6c), on the other hand, the oxidation of the electrolyte was reduced, thereby thinning the surface layer of the electrode. After 100 cycles, the lithium ions had already entered and exited the material multiple times, thereby the electrode surface also showed small cracks and reduced the agglomeration of the component particles of the cathode. On the surface of a single particle of the electrode that had undergone a large number of cycles, more cracks were be seen when the electrolyte did not contain the additive than when it did. It is speculated that the modified CEI film was flat, which effectively inhibited the decomposition of LiPF_6_ and thus prevented the generation of HF^31^. In the case of the cycled cathode in which the electrolyte contained no additive, the unevenness of the CEI film may have caused the irregular transport of lithium ions in and out of the material, leading to the generation of more stress and so of more cracks on the surface of the material particles.

**Figure 7:**
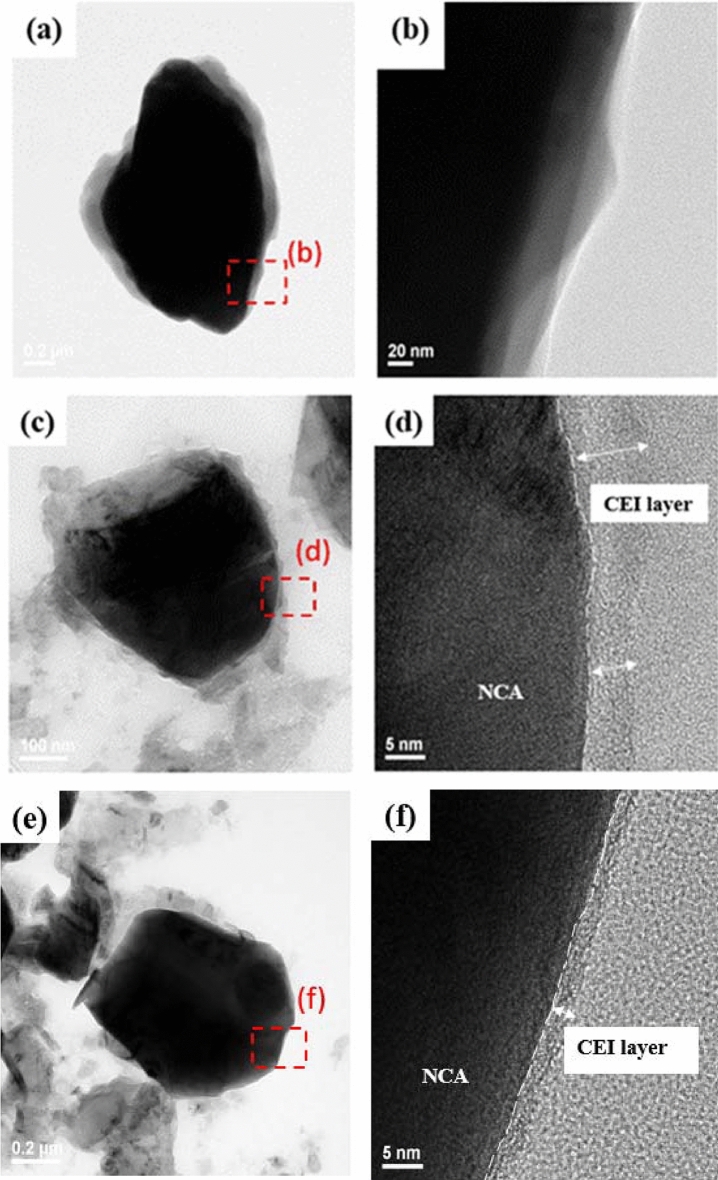
TEM images taken of the surface of the electrode: (**a**,**b**) a fresh NCA cathode, (**c**,**d**) a cycled cathode without ADM, and (**e**,**f**) a cycled cathode with 1 wt% ADM. The cycled electrodes were taken from coin cells after 100 cycles.

Figure 7 shows the transmission electron microscopy (TEM) images of the electrode both without cycling and after 100 charge/discharge cycles. The LNCAO cathode that did not undergo cycling exhibited smooth edges (Fig. 7a,b). However, an uneven and thick amorphous film was present on the LNCAO electrodes (Fig. 7c,d, for the electrolyte without ADM) as a result of the irreversible increase after multiple cycles and the decomposition of the organic electrolyte. The accumulation of organic compounds was uneven compared to that of inorganic compounds, indicating that the organic electrolyte without the additive underwent significant decomposition during cycling, which increased the impedance of the LNCAO in the CEI film. The uneven film allowed the easy transport of the lithium ions at a specific area, leading to the generation of stress and cracks in the material particles, as shown in Fig. 6b. With the additive in the electrolyte (Fig. 7e,f), the CEI film on the surface of the LNCAO cathode was thin, uniform and dense after 100 charge/discharge cycles, indicating that the additive prevented the organic electrolyte from decomposing. However, most of the inorganic compounds accumulated, forming a dense and stacked film which reduced the impedance of the LNCAO in the CEI film. The decrease of the irreversibility of the LNCAO electrode in electrolyte with ADM additive also reduced the thickness of the CEI film, making it more compact. The presence of lithium ions was not limited to a specific area on the transmission channel, which also reduced the occurrence of cracks in the particles^32–34^.

**Figure 8:**
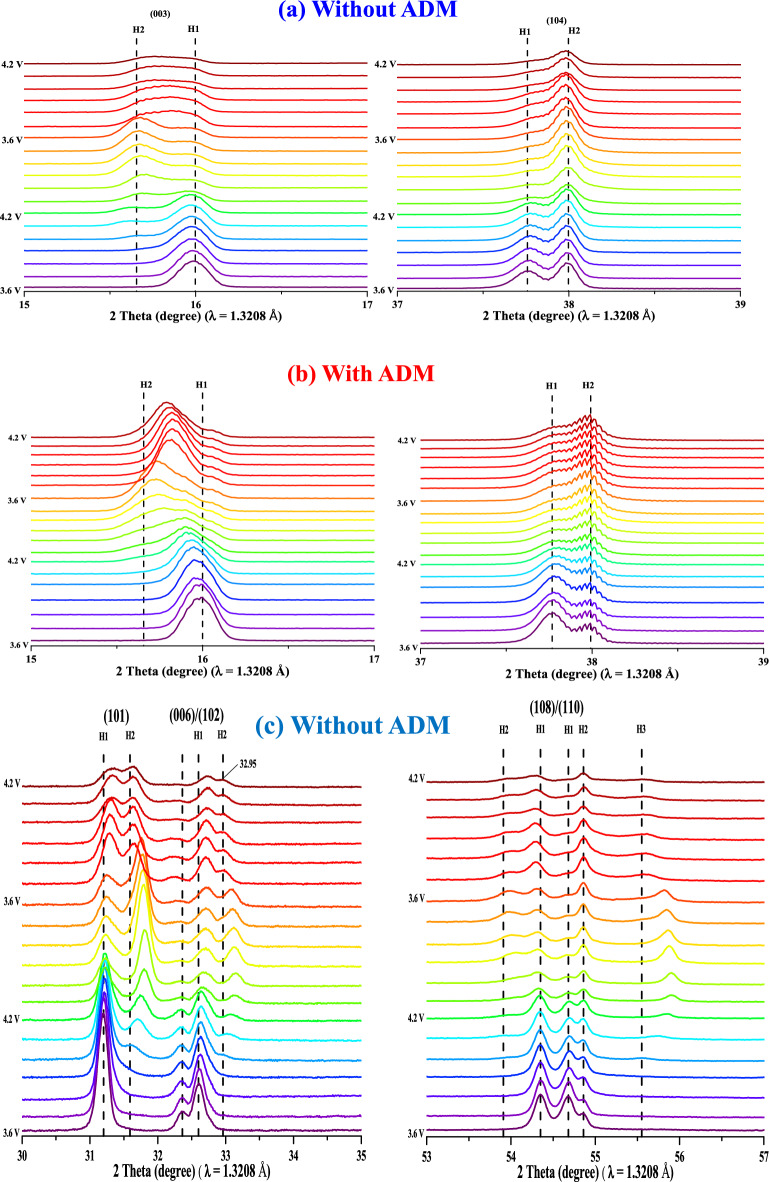

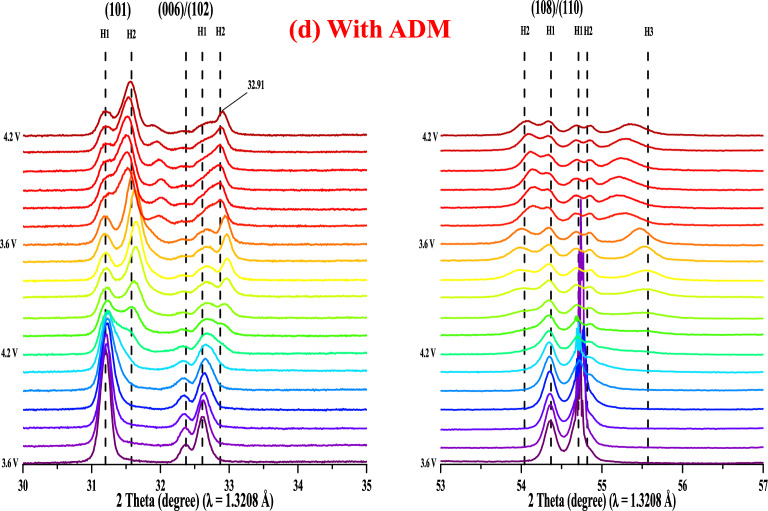
(**a**,**b**) In situ XRD (003) and (104) patterns for NCA electrodes (**a**) without ADM and (**b**) with ADM at different charge/discharge states from 3.6 to 4.2 V (H1 H2: hexagonal). (**c**,**d**) Selected in situ XRD diffraction patterns of the (101), (006)/(102), and (108)/(110) planes for NCA electrodes (**c**) without ADM and (**d**) with ADM at different charge/discharge states from 3.6 to 4.2 V (H1 H2 H3: hexagonal, M: monoclinic).

In order to evaluate the extent of the modification of the formation of the CEI film caused by the addition of ADM in the electrolyte, we investigated the structural changes of the LNCAO cathode by means of the synchrotron XRD technique (voltage range: 2.5–4.2 V, current rate is 0.15 C, and wavelength = 1.3208 Å). Figure 8 shows the XRD spectra corresponding to the (003) and (104) planes where the peaks splited into H1 and H2 (H1, H2: Hexagonal). The (003) peak shifted to a low angle, and the shift angle was small for the electrolyte with the additive (Fig. 8b). The reversibility was relatively high, indicating that the CEI film formed by the ADM effectively maintained the structural stability of the layered material. In addition, the (003) peak formed during the first cycle was relatively sharp in the absence of the additive (Fig. 8a) compared to the ADM-containing system (Fig. 8b), which indicates that the additive improved the interlayer phenomenon of Ni ions and Li ions^35^, thereby improving the reversibility of the lithium ions. Similarly, the (104) peak also exhibited splitting and shifted to a high angle due to the transition of Ni^3+^ to Ni^4+^, which compressed the a-axis and the b-axis of the transition metal layer, which is known as monotonic shifting^36^. Because of the phase change mentioned in the CV analysis, we know that there occurred an M phase transition between H1 and H2. However, the monotonic shifting of the (104) peak in the presence of the additive was less obvious, which means that the CEI film formed by the ADM effectively inhibited the conversion of Ni^3+^ to Ni^4+^, thereby maintaining the reversibility of the structure.

Figure 8c,d shows the diffraction patterns of the (101), (006)/(102), and (108)/(110) planes, with more obvious splitting of the (006) peak than of the others. The (101) peak gradually shifted to a high angle, corresponding to monotonic shifting because the conversion of Ni^3+^ to Ni^4+^ compressed the a-axis and the b-axis^36^. The peak displacement for the ADM-containing system (Fig. 8d) was less obvious, indicating an improvement in overall structural stability and reversibility. One of the indicators used to evaluate the layered material is the (006)/(102) peak, but the (006) peak did not show any shift, not even at 4.2 V during the second cycle. The H2 peak for the electrolyte without the additive (Fig. 8c) shifted to 32.95° compared to 32.91° with the additive, which was a minor change from the original H1 peak. This indicated that the ADM modification facilitated the formation of the CEI film during the first cycle and increased the stability of the overall material. Another indicator of the performance of the layered materials is the shift of the (108)/(110) peak from H1 to H2 in the absence of the ADM additive and the fact that the peak splitting was not obvious in its presence, indicating better structural stability.

**Figure 9:**
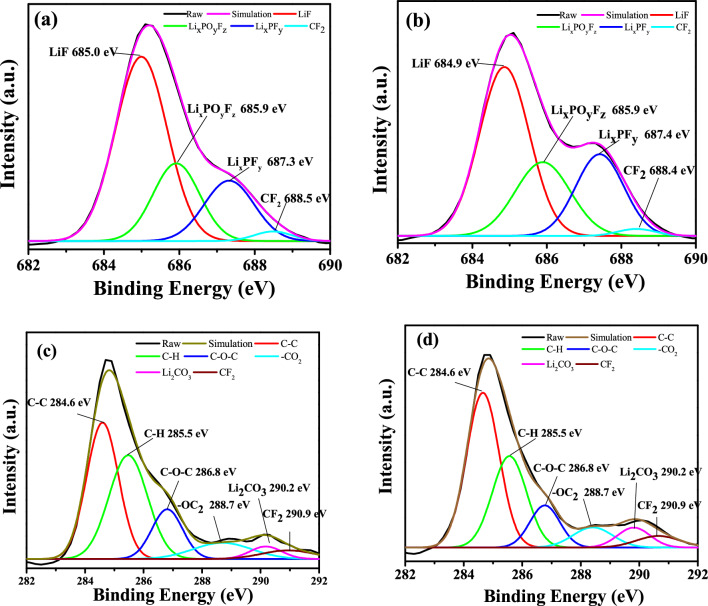
F1s XPS spectrum taken from the surface of the electrode: (**a**) a cycled cathode without ADM and (**b**) a cycled cathode with 1 wt% ADM. C1s XPS spectrum taken from the surface of the electrode: (**c**) a cycled cathode without ADM and (**d**) a cycled cathode with 1 wt% ADM. The cycled electrodes were taken from coin cell after 100 cycles.

Figure 9a,b shows the F1s XPS spectrum for the cathode after 100 charge/discharge cycles. The spectrum was deconvoluted^37^ to four peaks corresponding to LiF, Li_x_PO_y_F_z_, Li_x_PF_y_, and CF_2_ (approximately 865, 685.9, 687.3, and 688.5 eV, respectively) (see Table 3). Among these peaks, the major contribution came from the Li–F bond, which accounted for nearly 57.7% in the case of the electrolyte without the additive (Fig. 9a) and 50.5% with the additive (Fig. 9b), indicating a decrease in lithium fluoride caused by the additive in the electrolyte. On the other hand, the contribution from the Li salts (Li_x_PO_y_F_z_ and Li_x_PF_y_) reached 47.9% with the additive but only 40.1% without it, indicating that the presence of ADM effectively inhibited the decomposition of LiPF_6_ into LiF^13^. Finally, the CF_2_ bond of the PVDF accounted for a small part of the ESCA spectrum, at 1.6% for the electrolyte with the ADM and 2.2% without it. The C1s XPS spectrum (Fig. 9c,d) was also deconvoluted, confirming the contribution of the following bonds^38,39^: C–C, C–H, C–O–C, –OC_2_, Li_2_CO_3_, and CF_2_ (284.6, 285.5, 286.8, 288.7, 290.2, and 290.9 eV, respectively). The compounds having C–O–C and –OC_2_ bonds accounted for a high proportion of the spectrum for the cathode cycled without the additive in the electrolyte (Fig. 9c, Table 4), which means that the oxidation reaction in the organic electrolyte containing the EC and DEC caused the decomposition of the electrolyte. The compounds having C–C, C–H and Li_2_CO_3_ bonds accounted for a higher proportion of the spectrum for the cathode cycled with the additive in the electrolyte (Fig. 9d), indicating that the CEI film was thinner and so was more conducive to the transport of lithium ions. Therefore, under the influence of the ADM additive, the decomposition of the organic electrolyte was effectively inhibited because the value of the highest occupied molecular orbital (HOMO) of the ADM was higher than that of the EC and DEC^13^.

**Table 3:** ESCA data of F1s.

Assignment		Peak ratio	
		Without ADM peak ratio (%)	With ADM peak ratio (%)
Li–F	LiF	57.7	50.5
P–F	Li_x_PO_y_F_z +_ Li_x_PF_y_	21.8 + 18.3 = 40.1	24.3 + 23.6 = 47.9
C–F_2_	PVDF	2.2	1.6

**Table 4:** ESCA data of C1s.

Assignment	Peak ratio	
	Without ADM peak ratio (%)	With ADM peak ratio (%)
C–C	37.3	44.4
C–H	34.3	27.4
C–O–C	13	11.0
–OC_2_	8.1	7.0
Li_2_CO_3_	3.4	5.7
CF_2_	3.8	4.4

## Conclusions

We have investigated the electrochemical performance of an LNCAO cathode with an additive (ADM) in the electrolyte that facilitated the formation of a stable CEI layer at the cathode–electrolyte interface. The cathode delivered a good discharge capacity of 144.28 mAh g^−1^ (@ 100 cycles) at 40℃, with a high cyclic stability and a relatively good capacity retention (80%) and columbic efficiency (99.5%) compared to the electrolyte without the additive (37.5 mAh g^−1^, approximately 20%, and 90.4%, respectively). The ADM additive enhanced the structural stability of the surface of the cathode materials, whereas multiple cracks were observed when no ADM was added to the electrolyte. The ADM facilitated the formation of a thin, uniform and dense CEI film on the surface of the LNCAO electrode that can prevent the decomposition of the electrolyte and maintain the structural stability of the layered material.

## Supplementary Information

Supplementary Information.

## Data Availability

The datasets used and/or analysed during the current study available from the corresponding author on reasonable request.
