# Supplementary material for: Methylboronic acid MIDA ester (ADM) as an effective additive in electrolyte to improve cathode electrolyte interlayer performance of LiNi0.8Co0.15Al0.05O2 electrode
Source: Sci Rep. 2023 Jun 20;13:10025. doi: 10.1038/s41598-023-36341-8 (PMC10282062; doi:10.1038/s41598-023-36341-8)
Supplement: Supplementary file 1 — Supplementary Information. [file 41598_2023_36341_MOESM1_ESM.docx]

**Supporting Information**

|  | **R_S_ (Ω)** | | **R_SEI_ (Ω)** | | **R_ct_ (Ω)** | |
| --- | --- | --- | --- | --- | --- | --- |
|  | **Without ADM** | **With ADM** | **Without ADM** | **With ADM** | **Without ADM** | **With ADM** |
| 3^rd^ | 2.31 | 1.87 | 3.96 | 5.15 | 14.3 | 10.7 |
| 100^th^ | 3.75 | 11 | 16.2 | 10 | 163 | 75 |

**Figure S1.** Nyquist plots of LNCAO/Li half cells in 1M LiPF_6_ dissolved in EC/DEC (1:1) without/with ADM after (a) 3 cycles and (b)100 cycles
